# Extracts and Gleanings

**Published:** 1875-01

**Authors:** 


### Extracts and Gleanings.

---

*Principal Cause of Constitutional Derangement in First Dentition.* We subjoin the following extract of a paper read before the Iowa Dental Society, by Dr. J. Hardeman, and subsequently published in the Missouri *Dental Journal:*

"We maintain that the resistance offered by the gums is of but small moment. That the true cause arises from the pressure upon the nerve or pulp is certain; and that this pressure comes from an indirect way—namely, by the growth of the lower or root part of the tooth being too fast, so to speak, when compared to the osseous covering over its crown, thus holding it back, and itself thereby pressing unduly and irritating the pulp.

"This, then, is our theory, taking a cuspid as a case for illustration. The point is readily felt by the lancet in the hand of the surgeon; but his crucial incision in the soft tissues of the bone has but little effect upon the attending constitutional symptoms, and why? To answer, let us seek from whence this undue pressure.

"The point of the tooth having penetrated the bony surface, a large amount of resistance proper from below is taken off, hence allowing a greatly increased force to be exerted upon the borders of the socket opening, and this force or pressure being too great, is an irritant upon the portion of the capsule surrounding this opening, creating inflammation in it, and thus arresting absorption, holding the tooth, in other words, immovably fixed. While thus stationary, the growth of the tooth below still going on, must, it is obvious, produce indirectly pressure upon the pulp; and this is the whole mystery. Undue pressure augmented by the irritation and inflammation arresting absorption, fixing the tooth in the socket, while the steadily increasing tooth surely produces the pernicious pressure upon the pulp; and this, in turn becoming by this pressure irritated and inflamed, produces in remote organs, through the intimate nervous union before referred to, the extensive sympathetic disturbance so often met.

"A word as to the mode of local treatment in first and difficult dentition. The condition of the soft parts over the tooth are of little importance save in diagnosis. But the point in treatment is to relieve the tooth from its strangulation in the bony casement, and thus materially and effectually relieve the pulp from the undue pressure. To do this, we would suggest that with a spear-shaped chisel, or lancet-blade, the portion of the alveolus overlying and surrounding the impinged portion of the tooth be heroically broken up.

"But we would insist here that, to be effective, the surgeon should not hesitate to remove or break up the bony casement freely down or nearly to the largest diameter of the crown."

We have long felt the need of a more satisfying explanation of the multitudinous ills of teething children than the mere irritation of the gum, and if the theory above advanced be true, then have we gained something perhaps. Yet it seems scarcely reasonable that Nature would have been so remiss in the coördination of her powers as to permit a want of fitness between the tooth and its socket. Does not this view predicate local disease either in hypertrophy of the tooth, or in preternatural hardness in the resisting jaw-bone?

We are apprehensive, too, that the treatment proposed would, unless its virtue can beyond all shadow of doubt be established, prove rather too heroic for the approval of most of the mothers.— The subject, however, is an interesting one and worthy of consideration. A. A. L.

*Infant Diet.*—The *St. Louis Medical and Surgical Journal* says that, at a recent medical congress, held at Marseilles, the majority of the doctors present agreed that the best possible diet for children was oat-meal porridge and sweet milk. A. A. L.

*Diarrhœa of Typhoid Fever.*—The diarrhœa should be checked. The chalk mixture is good, but the chalk has to pass through many feet of intestine before it reaches the part desired. A better mode of treatment is to fill the lower bowel with a starch injection.

*The Claims of the Medical Profession.*—At a called meeting, in Boston, Massachusetts, of the patrons, friends and officers of Harvard University, for the purpose of devising the best method for collecting funds for erecting new and suitable buildings for the Medical Department of this old and honorable institution, several persons spoke in advocacy of the measure, and, among them, Dr. Oliver Wendell Holmes made a very able address. After considering various points of the subject, he concluded his speech with the following laudatory remarks *on the Claims of the Medical Profession on the Community.* There is no hyperbole, no effort, as it were, on his part, but no person can read what he says without feeling a glow of pride and a willing concession of all he claims for our noble profession.

We clip this from the *Boston Morning Journal* for October 23d, 1874:

"I come then to the claims of the medical profession on the community. Let me begin by quoting a passage from a recent writer who has said many plain, true and most unpalatable things to the clergy and the so-called Christian people of England—the author of 'Modern Christianity a civilized Heathenism.'

"'Men are pleased to call you *Reverend,*' he says, addressing the English clergy, 'but if such a title belongs to any profession on this earth, it belongs not to the parson but to the doctor. He it is who, in some degree at least, is making himself Christ to the suffering and the sorrowing among mankind. He it is who turns out of his bed at midnight to cool the poor man's burning lips, or succor a woman with the tenderest efforts of his skill, who can never pay him sixpence for his trouble, whether her infant lives or not.' 'What you do cheerfully enough once in a way, he does as a matter of business all day long. Your work is baby's play compared to his.'

"So writes a canon of the Established Church of England, if common report rightly assigns the authorship of that terrible satire.

"The physician's life is one of sacrifice. He gives up not only his ease, if necessary his health and even his life, but what is dearer to some men, I might almost say, than any of these—namely, his habits. He drops his novel with the last chapter unread; he leaves the theatre with the fifth act just working itself up to agony; he gets up from a meal that is untasted; he leaves his pillow unpressed, or springs from it in the dead of the night to brave the wildest storm of rain or snow; he has not an hour by night or day when you cannot summon him as if he were a slave and you were his master. He does more than the good Samaritan—he goes to the wayside to look for the wounded travelers and carries them in his ambulance to his hospital, which is an inn where there is no landlord to pay. He will stoop to wash your feet, if they are bruised and maimed, and do for you more than menial service at the call of humanity.

"These are his sacrifices—what are your gains? The surgeon is constantly saving life. Where would you be without his aid in a case of strangulated hernia? Think of those wonderful, and at first sight appalling operations—vivisections, I had almost said—by which hundreds of women have been rescued from inevitable death and come back to life, as the brother came forth from the sepulchre, as the maiden rose at the words of Him who said, 'She is not dead, but sleepeth!' And in woman's special hour of anguish, what do not she and those that love her often owe to the skill and care by which two precious lives are guarded or rescued? If the physician has not so often as the surgeon or the obstetrician the certainty that he has saved his patient from impending death, he cannot doubt that the measures he has taken not very rarely turn the uncertain balance in his favor.

"Most men want to live as long as they can and as comfortably as they can, and the great business of the physician is to help them in realizing both these wishes. I am not one of those whose tendency is thought to be to overrate the efficiency of medical treatment. I have been accused, on the contrary, of undervaluing some of the agencies employed in the treatment of disease. But while I never hope to see the great tidal movements of disease stayed by the employment of any drugs that we possess or are like to possess, I recognize with unspeakable gratitude the control placed in the hands of the physician over every form of suffering and discomfort. When a physician finds his patient panting, suffocating, drowning, in the fluid that is crowding his lungs, and boldly thrusting a hollow needle into his chest pumps it out and gives him his breath again; when he goes to a patient gasping with asthma, and pricking an atom of morphia into his skin so transforms him in the course of a few minutes that, to borrow a sufferer's words, whereas he had been in hell he was now in heaven; when he visits one who is undergoing the torture of the passage of a gallstone and silences the pain with an anæsthetic that says, "peace, be still," with an almost divine authority, I feel that nothing comes nearer to the Divinity than he who is invested with such beneficent capacities.

"The pains of surgical operations and of disease have been divested of much, if not of all, of their terror. The agony that seemed inseparable from maternity has been divorced from it in the face of the ancestral curse resting upon womanhood. With the first painless birth, induced by an anæsthetic agent, the reign of tradition was over, and humanity was ready to assert all its rights. It remains for the physician to claim for his art the right of procuring a painless passage out of the world, so far as is practicable, for the patient whom he can keep no longer in it, and without doing violence to the proprieties of the closing scene, to consider the physical process as one which should be under his exclusive direction.

A. R. K.

*Muriate of Ammonia for Nervous Diseases.*—Dr. D. J. Farnesworth, of Iowa, speaks as follows of the "Therapeutical Applications of Ammonia," in a lengthy paper read at the last meeting of the American Medical Association. We have frequently used it in such cases with decided benefit:

"Inhalation of muriate of ammonia is most decidedly beneficial, and affords very speedy relief in asthma and bronchitis. A bottle containing muriatic acid is connected with a bottle having a tube and mouth-piece; a third bottle containing aqua ammonia is connected with this. Inhalation through the mouth-piece brings the gases from each bottle, which unite in the tube. This method has been successfully tried in aphonia from cold or catarrhal affections. Nasal catarrh is also benefited by this treatment.

"As to the application of muriate of ammonia in nervous diseases, Dr. Anstie specifies them in the *Practitioner*, (December, 1868): 'Myalgia is speedily amenable to it in doses of grs. x. to xx. Various neuralgias are speedily cured, such as migraine, clavus hystericus and facial neuralgia. In intercostal neuralgia, especially in that form met with in nursing women or in phthisical patients, the remedy is of great value, frequently relieving the patient in half an hour. It may be used to advantage in the milder varieties of sciatica, which occur in young and debilitated patients, and in that somewhat obscure form of neuralgia termed hepatic.' He thinks it is the most powerful of all functional restoratives of suspended secretions. He especially recommends it in those cases where the disease is produced by severe and exhausting mental excitement.—He mentions several instances in which two or three doses of grs. xx. have caused a marked recommencement of suspended biliary secretions. He gives as a theory of its action that 'it is a pure tonic stimulant to sensitive vaso-motor nerves.' Dr. Farnesworth thinks, in these cases, it is the *stimulant* properties of the ammonia and the *alterative* properties of the chlorine which effect the good result."

A. R. K.

*Points in the Treatment of Diseases of Children.*—Dr. Fothergil read a paper in the Medical Society of London, reported in the *Medical Press and Circular*, in which he pointed out that, owing to the impressionability of the nervous system in children, depressants were only required for a brief time in the treatment of acute disease; that the plan of giving stimulants freely and habitually when in health was bad, but that in acute exhausting disease they might be used with benefit, especially in connection with easily assimilable food. Much mischief often arose from unfounded fears on the mother's part as to adding to the inflammation by such measures, and the consequence was that many sick children perished from lack of strength. He then alluded to the common condition of a ravenous appetite, co-existent with steadily progressive wasting; the more the child eats, the quicker it perishes of inanition, in consequence of its inability to digest food; restriction in the amount of food given often led to recovery. Diarrhœa in children was often a natural means of removing masses of indigestible or irritant material, and the plan of attempting to check such diarrhœa by astringents frequently led to a condition of enteritis. The treatment was to clear away the offending matter by a dose of rhubarb or castor oil, and then to change the dietary. In many children who were suckled beyond the weaning point, or when the food was insufficient, there exists a form of diarrhœa with green stools, which was curable by proper nutrition. Dr. Fothergill also thought that the anti-syphilitic treatment of disease in children who were congenitally syphilitic was confined too much to those manifestations which were associated with the pre-dental period of a child's existence. Experience had taught him that there were conditions of anæmia where the addition of mercury to the hæmatics administered was very beneficial, up to the time of the second dentition, if, indeed, that or any other limit could be assigned. The diathetic element required its special treatment as much in congenital syphilis as did the cachexia of acquired syphilis for successful practice. Finally, he drew attention to the conditions of excessive acidity of the secretions of strumous or scrofulous children, to the sour perspiration, the destruction of the teeth, and the uric acid in the urine. This might be due to imperfect oxidation, or imperfect nutrition and assimilation, or both combined. As to the first, we are all familiar with the excellent effects of fresh air, especially at the sea-coast, upon strumous children, and Sugol had found such children much improved at harvest-time when out of doors, gleaning. Alkalies, especially potash, had been found useful by Brandish, Brodie, and others, as removing or neutralizing the excessive acidity; but, in addition to this, rectification and improvement of the nutritive and digestive processes were necessary. It was possible that many of the peptones never became tissue, but were directly oxidized into urine products, and also that more sugar was split up into lactic acid than the system could oxidize, and so it accumulated in the body.

*Acetic Acid in Mucous Polypus.*—Dr. Méplain, of Moulins, gives the history of a man, thirty years of age, who had been afflicted for a month with mucous polypus of the velum palati. The tumor was growing rapidly and impeded deglutition and speech, and gave rise to considerable hemorrhage. Applications of chromic and carbolic acids, removals with scissors, etc., afforded no relief, the tumor being constantly reproduced. A drop of acetic acid was injected into the polypus, and it immediately began to shrink; a second injection completed the cure, and five months later there were no symptoms of a return of the disease.—*Bull. de Thérapie* and *Gazz. Med. Ital. Prov. Venete,* No. 9, 1874.

*A New Method for Healing Ulcers.*—Dr. Nussbaum (*Phil. Med. Times* from *Wien Med. Presse*) claims to have successfully treated upwards of sixty cases of chronic, extensive, and otherwise intractable leg ulcers, by the following simple procedure:

The patient is at first etherized, and then around the ulcer of the leg or foot, a finger's breadth from its margin, an incision extending down to the fascia is made; numerous blood-vessels are divided, and a severe hemorrhage ensues, unless a fine pledget of lint be packed into the cut and the entire ulcer strongly compressed. The packing with lint is also necessary to prevent union of the cut edges by the following day. Upon the second day the bandage is removed; from then until a cure is effected, a simple water dressing is applied.

The author states that an astonishing change can be seen, even in the first twenty-four hours. The ulcer which yesterday threw off quarts of thin, offensive, ichorous pus, furnishes to-day not more than a table-spoonful of thick, non-offensive, healthy pus. The old ulcer becomes rapidly smaller, healing from the margin towards the center, and is healed up in a short time; but the cut is changed into a broad circular sore, which also rapidly cicatrizes.

The great diminution of the secretion, and other favorable changes occurring in the ulcer, find an explanation in the fact that the circumcision has divided dozens of large, abnormally widened blood-vessels. Time is thus given for the lessened nutritive material, which was previously carried off by the excessive secretion, to be transformed into cells and connective tissue; in other words, granulations are formed, which fill up and heal the deep ulcer. Without claiming this as a radical method, the author assures us that the cure is much more rapid, and the cicatrix becomes more elastic and resisting, than in ordinary means applied, which usually require so much time that the patients depart with half-cured ulcers, soon finding themselves in their previously deplorable condition.

[The practice of making incisions in the sound tissue bordering on diseased parts, for the purpose of promoting the more healthy action of those parts, is not new. It was employed by the late Professor E. W. Cooper, of San Francisco, though we cannot say whether it was original with him or otherwise.—*Ed. Pacific Med. and Surg. Journal.*

*Hydrate of Chloral as a Solvent.*—Mr. Robert F. Faithorne writes to the *American Journal of Pharmacy:*

A solution consisting of nine parts of hydrate of chloral and three of water, I find capable of dissolving the following substances, to the extent named:

One grain of morphia is dissolved by a portion of the liquid containing twelve grains of the hydrate, one grain of veratria by a portion containing five grains, and one grain of atropia by a portion containing twenty grains.

These active principles should be in powder, mixed with the solvent in test-tubes, and heated by means of a water-bath, with occasional agitation.

The solutions thus made are in a convenient form of employment, either alone or when mixed with oils, ointment, or with glycerin. Camphor, too, is freely dissolved by them, and in some cases can be added to them with advantage.

Glycerin I find to be a convenient agent for forming solutions with the chloral and the above named substances, and the following will be found, when properly combined, to produce permanent and elegant preparations, viz:

*Chloral Glycerite of Morphia*—℞. Morphia (powd.), gr. v.; chloral hydrate, ℨj.; glycerin, fl. ℥ss. M. Sec. art.

*Chloral Glycerite of Veratria*—℞. Veratria, gr. v.; chloral hydrate, ℨj.; Glycerin, fl. ℥ss. M. Sec. art.

*Ointment of Chloral and Veratria*—(Corresponding in strength to the Ung. Veratriæ, U. S. P.) ℞. Veratria, gr. x.; chloral hydrate, ℨj.; water, 6 drops; lard ointment, ℥ss. M. Sec. art.

*Chloral Glycerite of Morphia and Camphor*—℞. Morphia, gr. v.; chloral, camphor, aa ℨj.; glycerin, fl. ℥ss. M.

*Lotion of Chloral and Iodine*—℞. Iodine, gr. xx.; iodide of potassium, gr. vi.; glycerin, fl. ℨj.; chloral hydrate, ℨij. M. Sec. art.

Chloral can also be combined with collodion, in which it dissolves after the addition of a few drops of alcohol.

*For Incontinence of Urine in Paralysis.*—℞. Morphiæ sulph., veratriæ, aa. grs. v.; axungiæ, ℨj. M. Rub well into the perineum three times daily.

*Beneficial Effects of Vaccination.*—The Registrar-General of Scotland, in his last annual report, makes some observations on vaccination which are important. The beneficial efficacy of vaccination he attests by some striking statistics. He says:

"Before the introduction of vaccination into Scotland, from 12 to 14 per cent. of the total deaths were annually caused by small-pox, while nearly 2 per cent. of those who survived its ravages lost their eyesight, and a very large proportion had their countenances disfigured for life. Since vaccination was introduced into Scotland, in 1799, the average annual death-rate from small-pox, up to the present day, has been only $1\frac{1}{2}$ per cent. of total deaths, and even that number has chiefly been caused by the deaths of persons who never had been vaccinated. This single fact proves of itself, more convincingly than any arguments, the saving of human life which the general adoption of vaccination has effected."—*Medical Times and Gazette.*

*Keeping the Bed After Confinement.*—Dr. William Goodell, of Philadelphia, in his account of the arrangement of lying-in women in the Preston Retreat, writes as follows in regard to the common practice of keeping the bed:

"Lying-in women are encouraged to get up for food when they feel so disposed, because there are, to my mind, strong objections to the rigorous maintenance of the recumbent posture. Labor is, in general, a strictly physiological process, and there can be no sound reason why it should be made to wear the livery of disease. Nature teaches this very plainly, for most women wish to get up long before their physicians are willing to let them. The fact of a woman's wishing to get up is, to me, a very good reason why she should get up. In the second place, few physicians will deny that nothing so relaxes the tone of muscular fibre as a close confinement in bed. In my experience, a woman ordinarily feels stronger on the fifth day than she does on the ninth, if rigorously kept under quilts and blankets. Once more: the upright position not only excites the womb to contract, but, by distributing the blood and equalizing the circulation, it actually lessens the amount of the lochia and shortens their duration. On the other hand, the dorsal deubitus keeps up a passive congestion of the womb as a whole, the engorgement of the greatly hypertrophied placental site, and a blood-stasis in the now thickened posterior wall—all important factors in hindering the procees of involution. Again: uterine diseases are hardly known among those nations whose women early leave their beds. From passages in the writings of the classics, it is evident that, among the ancient Greeks and Romans, those models of physical strength and beauty, the women arose, and even bathed in a running stream, very shortly after delivery; in some cases on the very day. Finally, what is sounder than all theory, a sufficiently long and well-sifted experience has proved to me that, by such a treatment, convalescence is rendered far more prompt and sure. At this result, very unexpected to the multiparous patients of the Retreat, they are constantly expressing their surprise.—*Pacific Med. and Surg. Journal.*

*Diphtheria.*—A writer lauds the pinus canadensis in the treatment of this disease. He says: I made use of this remedy as follows: ℞.—Conc. fluid ext. pinus canadensis, ʒ ij.; aqua pura, ℥ viii. Sig. One teaspoonful taken into the mouth, allowing it to remain a few minutes, and then to be injected; immediately after which the same quantity was to be swallowed. This could only be done by great effort, and the holding of the head in a position which favored the process of swallowing; this to be repeated every half hour until four doses of each have been taken, after which it was used every hour. In three hours from the time the first spoonful was swallowed, a change was manifested. The patient was able to swallow beef-tea without so much pain and difficulty. In fifteen hours from the time the first dose was given, there was not a particle of the coating on the tongue to be discovered. The appearance of the ulcers was favorable, and the nasal obstructions were entirely removed; the alarming symptoms subsided, and convalescence was fully established; recovery was rapid. I continued the use of the medicine, reducing its strength one-half, and administering it at intervals of two hours for ten days, at which time all medicines were discontinued. I will repeat that at the time of beginning the use of this medicine, the strength of the patient was so completely exhausted that she was unable to rise from her bed or to stand upon her feet. Without conjecturing why this agent, possessing in its chemical constitution the same properties contained in many other remedies, should be more successful, I must cordially recommend this preparation to the careful consideration of the medical profession, especially in the treatment of this form of the disease.

Since writing the above, I have treated other cases presenting similar characteristics, with like results.

*The Treatment of Phthisis.*—The treatment of phthisis, according to Dr. Pietra-Santa, of Paris, can be summed up as follows : 1. Hygienic measures to be strictly attended to throughout the different stages of the malady, such as pure air, good substantial food, tonics, moderate exercise, milk diet, etc. ; 2. To utilize the changes effected in the organism by the mineral waters (sulphurous, arsenical, chlorides ) ; 3. To take advantge of the salutary effects of change of climate by a residence in the temperate climates of the South of Europe in Winter, and mountainous countries in Summer ; 4. To neutralize the morbid ferments engendered in the organism by purulent absorption, which takes place during the period of the *ramollissement* of the tuberculous matter. This, according to M. Pietra-Santa, is best effected by the administration of the alkaline and earthy hyposulphites and sulphites. 5. Complications which are inseparable from the affection ought to be treated according to the indications present.—*Med. and Surg. Reporter.*

*Nicotine as an Antidote to Strychnia.*—Professor Haughton, of Dublin, relates a case of strychnia poisoning which was treated by drop doses of nicotine in whisky punch every half-hour. At the second dose the paroxysms became less violent, and the muscles of the abdomen softer. Four doses produced relaxation of the muscles. This confirms previous experience on this subject. Dr. Fraser, Professor of Materia Medica at Aberdeen, says that nicotine combines a feebly paralyzing effect on the motor nerves, with a more decided paralyzing action on voluntary muscular fiber. The action of nicotine very much resembles that of physostigma (or calabar bean), acting, however, more directly upon the muscles, and producing complete relaxation of them. Physostigma acts by diminishing, and in large doses destroying the reflex activity of the spinal cord.—*British Medical Journal.*

*Iodoform in Granular Lids.*—Dr. O. P. Barber says: Without going into any "profound analysis of the subject," allow me to tell what I know of treating granular sore eyes with iodoform.

Some time ago I had a patient who had sufferd much from many physicians, myself included.

He was a sneaking, sniveling son of a gun, and would come siding into the office with a drooping, mournful expression in his large, sore eyes that was truly aggravating.

Months passed by, and still my sore-eyed friend haunted my office with such punctuality and pertinacity that I resolved to cure those eyes or die.

I brushed them with all the remedies known to modern occulistic surgery, and floated his intestinal apparatus in a sea of tonics and alteratives; and when all these things had failed, I used to take him around to medical societies as a sort of permanent clinique, and then to my office and try the suggestions; but the patient grew worse gradually, and so did I.

Finally, in a medical journal I saw an accouut of the successful treatment of an intractable ulcer of the leg with iodoform, and the idea suggested itself that it might be "good for sore eyes," and I had twenty grains put in an ounce of glycerin, and applied some to the inner surface of the lids, and in came the patient next day, with a sweet smile and a whitening cornea, and I felt as "if my life's work was ended"—that I could "step down and out."

The patient got well without a drawback in a short time, and I have tried the remedy in quite a number of cases since, with very gratifying results.

*Gurjun Oil in Leprosy.*—Dr. J. Dougall, of Port Blair, Andaman Island, has published a report of his experience with gurjun oil in the treatment of leprosy. Of twenty-four cases under treatment during a period of six months, he says that all have been decidedly benefited. The oil is administered internally in combination with lime-water, and is also applied externally to the affected parts. Gurjun oil is an oleo-resinous product of the *Dipterocarpus lœvis* and other allied trees. Experiments with the new remedy are being made at the present time in St. Bartholomew's Hospital, London, and elsewhere.

*"Persistent" Inflammation of Joints—Oleate of Mercury and Morphia.*—When pure oxide of mercury, freshly prepared by precipitation, is added to oleic acid, it combines with it, forming an oleate of mercury dissolved in oleic acid. A small amount of pure morphia (gr. i. to ʒj.) should also be added. A solution containing five per cent. is limpid and yellow, ten per cent. dark, and 20 per cent. more of the consistence of an ointment, melting at the temperature of the body. Let the five per cent. solution be brushed over an inflamed knee-joint which has resisted other means of cure, and the mercury and morphia will be rapidly dissolved, arresting the inflammation and relieving the pain. As a rule, according to the size of the part affected, from ten to twenty drops are sufficient for one application. This should be repeated twice daily for four or five days, then at night only for four or five days, and afterwards every other day until a cure is obtained. The diffusibility of oleic acid is much greater than that of ordinary oils or fats. The knee is merely taken for illustration; the same plan is equally efficacious in inflammations of other external parts of the body.—*Professor J. Marshall.*

*Typhoid Fever.*—Animal broths and jellies must be rigidly excluded from the diet. Milk should be the chief article of diet, having a little ice added to it if agreeable, and a little lime-juice in cases in which it returns curdled. Rennetted milk, rice milk, custard, rusks and hot milk, or blanc-mange, generally afford sufficiently varied ways of giving milk. Two or three cups of really good tea or coffee should be given between daybreak and two in the afternoon, every day, unless there is some positive contra-indication in the state of the nervous system. Dr. Parkes found that coffee increases the elimination of urea in fever, and there is no doubt that both it and tea lessen drowsiness and prostration. If early in the fever there is flushed face and headache, a high temperature, and thickly-coated tongue, four or six grains of calomel should be given. Ergotine administered hypodermically is the best remedy in case hemorrhage from the bowels occurs. The following is a good pill to check the excessive action of the bowels: three grains of bismuth, one-sixth of a grain of opium, and one-sixth of a grain of carbolic acid.—*Dr. J. Little.*

*Erysipelas.*—Dr. J. J. Chisolm (New York *Medical Record*) says: Believing the glossy, red, diffusing swelling to be only the local manifestation of a constiutional disorder, I have for many years restricted the treatment of erysipelas to internal remedies, ignoring, as far as medication is considered, the local condition. Experience has taught me to place every confidence in the use of iron, as nearly antidotal to erysipelatous poisoning. I administer in all cases the tincture of the muriate of iron, as soon as the complication is diagnosed. Formerly I thought it necessary to prepare the system for the administration of the iron, by first giving a mercurial purge. For the past fifteen years I have disregarded this preparation, using the tincture of iron alone from the beginning to the end of the treatment. The dose of the iron is from half a drachm to a drachm every four hours. With such doses I have often cut short what threatened to be a serious attack of traumatic erysipelas in forty-eight hours. The idiopathic variety will also yield promptly to this treatment, with scarcely an exception.

*Lacto-Phosphate of Lime in Mineral Inanition.*—The importance of phosphate of lime in the animal economy has scarcely been hitherto appreciated. So necessary is it that, if deficient, the tissues even draw from the osseous skeleton that which is necessary for the maintenance of the integrity of the functions of nutrition. If animals are artificially deprived of phosphate, there is a loss of appetite and general debility. Ordinary phosphate of lime is soluble to a great extent in a healthy stomach, but when the digestion and vital power of the system generally is impaired, it becomes dissolved with difficulty, forming a lacto-phosphate of lime, which is easily assimilable. The most convenient forms for administration are Dusart's Lacto-Phosphate of Lime Syrup and Wine. Fifteen grains of the lacto-phosphate is contained in each table-spoonful of syrup.—*Dr. Blache.*

*Why do we give Wine in Fever?*—Not because the tongue is brown or moist, not because there is delirium, nor on any other account than because the state of the circulation requires it. Alcohol is the remedy for failure of the heart's power, and this may occur with or without delirium, and with a dry brown tongue or a moist one.—*Dr. L. S. B.*

*Iodic Acid in Hypodermic Injections.*—Dr. Luton has been investigating the above substance, the properties of which appear to be very remarkable. Iodic acid is highly soluble in water; a solution to one-fifth has been obtained, and it is that which Dr. Luton employs. In this proportion, iodic acid does not produce a sore, but it causes amidst the tissues into which it is injected a deep modification which induces rapid absorption. Dr. Luton has employed it in goitre, in indolent adenopathic swellings of the servical and sub-maxillary regions, and in case of osteo-periostitis of a phalanx of the hand. The results have been excellent. Upwards of half a drachm of the solution, as above, has been injected at once. This substitutive injection is thrown into the midst of the tumor, and Dr. Luton thus utilizes the natural envelope of the ganglion or degenerated growth, and avoids a diffusion which might be attended with some inconvenience. The local reaction consecutive on the injection is somewhat marked, but is never accompanied by an accident; resolution, without suppuration or mortification, is almost invariably the rule.—*London Lancet.*

*Cardiac Disease.*—Cardiac disease before forty is more frequently on the left side of the heart than the right, and is the result of inflammatory change; after forty, disease is more frequent on the right side than the left, and is the result of tissue degeneration.

Degeneration of the tissues of the body begins at forty. Degeneration of the muscular structure of the heart is especially predisposed to by the habitual respiration of impure air in closely-shut, non-ventilated, hot, stifling rooms, whether in private houses, public buildings, theaters, schools, or manufactories.—*Dr. C. Black.*

*Hiccough and Spasmodic Affections—Oil of Amber.*—Oil of amber is an antispasmodic of considerable value in cases of hiccough and other spasmodic affections. In the Pharmacopœia of 1809 it was introduced, combined with ammonia as "spiritus ammoniæ succinatus." In a case of severe cramps in the limbs during pregnancy, the following was the prescription used: ℞. Olei succini, ℨss.; liq. potassæ, ℨj.; tinct, camph. co., ℥ss.; aquæ menth. pip. ad ℥vj. M. An ounce to be taken at bed-time.—*British Medical Journal.*

*Black Cohosh in Rheumatism.*—In rheumatism, I have found the cohosh a useful article, in both the acute and chronic forms. In over one hundred cases of acute rheumatism, I have never failed of curing when called upon the first attack of the disease, by administering sufficient of a saturated tincture of the root (from three to sixty drops), every two hours night and day, until the head becomes quite affected; then lengthen the intervals between the doses to three, four or six hours, sufficient to keep up the action on the brain, and which must be continued for not less than seven days, or until the disease is completely removed. Previous to the exhibition of this tincture, the bowels must be cleansed by a purgative, which may be occasionally repeated. This appears to change the rheumatic diathesis, so that a second attack will seldom occur.

In all attacks subsequent to the first, I employ the following tincture: Saturated tincture of black cohosh, two ounces; saturated tincture of nux vomica, one ounce. M. Commence, as a general rule, with ten drops, three or four times a day, and gradually increase to sixty drops. Or the following may be substituted, which is also beneficial in chronic rheumatism: Take of finely powdered black cohosh root, two ounces; finely-powdered pokeroot, two ounces; finely-powdered prickly ash bark, two ounces; finely powdered blood root, half ounce; alcohol, two pints. Let these stand for fourteen days, frequently shaking; from thirty to sixty drops, three or four times a day.—*Eclectic Med. Jour.*

*Puerperal Mania—Chloral Hydrate.*—In a case of puerperal convulsions terminating in mania, in which there had been no sleep for five days, and in which, during that period, there had been not the least cessation of the maniacal symptoms, twenty-five grains of chloral procured a refreshing sleep of three hours, and a repetition of this treatment for a few nights effected a complete recovery.—*Mr. F. V. McDowell.*

*Remedy for Chronic Hoarseness.*—In chronic hoarseness arising from thickening of the vocal cords and adjacent membrane, the ammoniated tincture of guiacum is often a very efficacious remedy. It may be appropriately mixed with equal parts of the syrup of senega, and a tea-spoonful of the mixture given two or three times a day.—*American Practitioner.*

*Positions for Catheterism.*—Mr. Teevan, Surgeon to West London and St. Peter's Hospitals, says, in the London *Lancet*, that the sitting position is best for the surgeon, and the standing best for the patient. The following are some of his reasons:

"However prolonged the operation may be, the surgeon will not become fatigued. . . . It is in the one position in which he enjoys the maximum amount of mobility with the minimum of power, and is therefore the one position in which he is least likely to make a false passage. . . . If the surgeon adopts the sitting position, I feel convinced that he will, in the long run, prove a far more successful operator than one who stands, and that he will often succeed where the latter had failed."

In the patient, "what we desire is fixity, and we shall best achieve this object by placing him upright against the wall and stretching his penis horizontally forwards. So situated, we have at last obtained all that can be desired; for the urethra, instrument, and direction of force are all in the same horizontal and vertical plane, and our axis of vision will nearly correspond. Thus, therefore, we shall be able to impress on the point of the catheter, when lost to view, any direction we may desire it to seek, and our operation will no longer be an exhibition of blind and unguided force, but a successful demonstration of applied skill."

*For Uterine Neuralgia.*—℞. Tinct. aconiti rad. (Fleming), ʒiss.; ammonii chloridi, ʒij.; ammonii iodidi, ʒi.; tinct. card. comp., ℥i.; syrugi auranti, ℥iv.; aquæ anisi, q. s. ad. ℥viij. M. Sig. Tea-spoonful every four hours. Also give, half an hour before each meal, a tea-spoonful of the syrup of the phosphates of iron, ammonia, quinia and strychnia. This treatment seldom fails to give relief after all other means have failed.

*Formula for Intertrigo.*—M. Legal recommends the use of subnitrate of bismuth in eruptions caused by scratching, induced in turn by the itching of intertrigo. Glygerin is employed as an excipient, because it does not become rancied, and the tincture of cochineal to give a color. ℞. Subnitrate of bismuth, glycerin, of each, eight grammes; tincture of cochineal, twenty drops.—*American Practitioner.*

*The Administration of Phosphorus.*—In the *Practitioner*, Mr. J. A. Thompson gives the following formula for the administration of phosphorus: ℞. Phosphorus, gr. j.; absolute alcohol, f℥ v.; glycerin, f℥ iss.; spirits of wine, f℥ ij.; spirits of peppermint, ℈ ij.—One drachm of this mixture contains one-twelfth of a grain of pure phosphorus. This combination possesses little or no phosphoric odor, and, according to Dr. Radcliffe's theory, "that the disappearance of the characteristic odor from a solution of phosphorus is presumptive evidence of the oxidation of the drug," ought to be inert; but Mr. Thompson's successful results in thirteen patients show that this is not the case. Mr. Thompson has given the phosphide of zinc a trial in six cases. This compound is prepared by bringing phosphorus vapor into contact with melted zinc in an atmosphere of dry hydrogen. Only one-half the phosphorus in this substance is said to be available for therapeutical purposes; two-thirds of a grain of zinc phosphide corresponds to one-twelfth of a grain of phosphorus, ard Mr. Thompson finds this the most efficient dose. The drug may be given in the form of pills, in combination with other substances, *e. g.* quinine, aloes or strychnia, and these pills are found to keep well. The remedy is not so speedy in its action as the solutions of phosphorus in oil or alcohol. Of the six cases treated with the phosphide, there was perfect recovery in two, and two were remarkably relieved.

*Compound Syrup of Garlic.*—℞. Fluid ext. allii, ℥ ij.; syrupi scillæ, ℥ iv.; tinct. opii comp., ℥ ij.; syrypi simplicis, ℥ ij. M. A tea-spoonful is a dose for a child, three or four years old, for catarrh. This prescription has been renewed over a hundred times in one drug store here, during the last four years.—*Dr. J. W. Robbins, Philadelphia.*

*Pyrosis.*—Iodine is a remedy of unquestionable efficacy in checking that kind of vomiting which constitutes what is called pyrosis. Three to five drops of the tincture should be given in an ounce of water. Although this remedy is of value, nitrate of silver, given in pills, immediately after food, is perhaps more trustworthy.—*Dr. J. K. Spender, British Medical Journal.*

*Causation of Typhoid or Enteric Fever.*—[Dr. E. M. Snow, the able and experienced Health Officer of Providence, R. I., in his report, makes the following remarks on the causation of typhoid fever, which are in full accord with our own experience, and we believe with that of physicians generally in California.—*Ed. P.M. and S. Journal.*]

"It is a common opinion, very frequently stated and very generally accepted, that the foul emanations from sink-drains, cess-pools and privy-vaults are one of the most important, if not the chief cause of typhoid fever. My observation does not confirm this theory. The disease prevails much the most in the country, where, if these emanations exist, they are largely diluted by the free circulation of air, while thousands in the city who breathe the foul air from these sources constantly are *comparatively* exempt from it.

"Again, the cases of typhoid fever in this city are as often in the comfortable and cleanly dwellings as in the poor and filthy. Of the nine decedents in September, five were of American and four of foreign parentage; of the six hundred and sixty-seven decedents from typhoid fever in seventeen years, 1856–1872, there were three hundred and seventy of American and two hundred and ninety-seven of foreign parentage.

"Again, I have known some marked instances of severe typhoid fever evidently caused by decomposing vegetable matter, with no aid of sink-drains or other nuisances. In one instance, several families in one house had typhoid fever, apparently caused by thirty bushels of rotten potatoes in the cellar.

"I think that typhoid fever is caused chiefly by the decomposition of vegetable substances, and that for this reason it is far more prevalent in the country than in the city."

*Delirium in Typhoid Fever.*—In the treatment of this complication remember that it is owing to brain-irritation, not to inflammation. There is one great remedy for this, and it is alcohol; alcohol is the remedy for nervous irritation in typhoid fever, and, in fact, in any fever. How it acts is not certainly known, but it may be laid down that it is an important point in the treatment of all fevers. It allays nervous irritation and soothes the nervous system.—*Sir W. Gull.*

*Chloroform in Strychnine Poisoning* (*The New York Medical Record*).—A man took five grains of strychnine with a suicidal intent. He was given twenty grains of the sulphate of zinc, which produced vomiting. Convulsions had occurred repeatedly, however, and he was seized with one of tetanic form at the time of coming under observation. Every muscle was rigid, and tetanus was complete. Opisthotonos, irregularity of the pulse, varying from 120 to 140 in the minute, with all the accompanying symptoms, were noticeable.

He was immediately placed under the influence of chloroform. The convulsions ceased from the commencement of the anæsthesia, under which the patient was fully kept for three hours. The chloroform was then removed, but the patient did not awake until six hours afterwards—a case of recovery.—*Medical Examiner.*

*Digitalis.*—M. Gaunot, in the course of a paper published in the *Gazette Medicale de Paris,* on the action of digitalis, says: "When digitalis or digitalin is administered for some time to a man in full possession of sexual powers, these become gradually weakened, the propensities disappear, formation of the liquor seminis diminishes, and may at last cease altogether. The anaphrodisiac properties of the drug are the secret of its good effect in spermatorrhœa."

Dr. Little recommends the use of digitalis in febrile cases in which stimulants are either not well borne or are contraindicated, as where renal affection is present. He has given half-drachm doses of the tincture every three or four hours in typhus, enteric and rheumatic fevers, with very favorable results.—*Edinburg Medical Journal.*

*Eczema Capitis.*—Cure it if possible, notwithstanding the somewhat prevalent idea to the contrary. The method of procedure recommended is as follows: First, apply a poultice every night until all the scabs are removed. The ulcerations, which are sometimes present after the scabs have been removed, are best cured by the application of a wash of nitrate of silver grs. v. to the ℥i. of water. The following is then used with good success: ℞. Aquæ, ℥iv.; glycerin, ℥ij.; carbolic acid crystals, ʒi.; borax, ʒi. M. Continue the application of this remedy for some time, for the purpose of curing eczema.—*N. Y. Med. Record.*

*External Use of Turpentine in the Treatment of Tonsilitis.*—In the *Leavenworth Medical Herald* Dr. S. H. Roberts strongly recommends the use of turpentine externally in tonsilitis. He folds the flannel to four thicknesses, wrings it out in hot water, and pours oil turpentine over a spot the size of a silver dollar. The flannel is then applied over the sub-parotid region, and the fomentation continued as long as it can be borne. After removal, a dry flannel is applied, and the same region rubbed with turpentine every two hours. This application is continued daily till resolution occurs. The doctor believes, from the evidence of his long experience, that thus applied early in the disease the oil of turpentine has almost a specific effect in tonsilitis. That its action is not simply that of an irritant, he has proved by employing mustard, croton oil, tr. iodine, etc., in the same class of cases. They always failed to diminish the inflammation of tonsils, while the turpentine succeeded.—*Detroit Review of Medicine.*

*Ulceration of the Vagina, Larynx, and other parts, in Typhoid Fever.*—There may not be only the ordinary ulceration of the ilium in cases of typhoid fever, but ulceration of other parts. A young girl was sickening of an illness. She had a discharge from the vagina, which led the medical attendant to doubt the girl's chastity; the case was really one of typhoid with ulceration of the vagina. Another case was treated for gonorrhœa; but in reality the patient had typhoid fever with ulceration of the prepuce. Another patient had ulceration of the larynx in the course of typhoid fever; he suddenly became emphysematous.—*Sir W. Gull.*

*Oat Meal Farina as a Food for Infants.*—MM. Beaumetz and Hardy recommend very highly the use of oat meal farina in the feeding of young children. According to these gentlemen, this farina resembles human milk most closely in its plastic and respiratory elements, and contains, in addition, iron and phosphate of lime. It has, besides, the property of preventing or arresting the diarrhœa, which so frequently occurs in young children. Some infants of four to eleven months who were fed npon this farina, were found to grow equally well with those who were nourished by the milk of a good nurse.—*Medical Examiner.*

*Anointing with Cocoa Butter in Scarlet Fever.*—Upon the recommendation of Schneeman, the anointing of the body with fat has been extensively practiced in Germany during the past ten years, with the view of lowering the temperature and hastening the desquamation. Dr. Bayles suggests, in this connection, the employment of cocoa butter, as producing a more cooling and refreshing effect upon the patient, and emitting a more agreeable odor in the sick chamber. This agent, on account of its solid consistence, is more readily applied than either fat or oil, and is more easily absorbed by the skin. Furthermore, it is thought to afford the system a certain amount of nourishment.

In severe fevers, the entire surface of the body should be rubbed with this substance every hour, or at least once every four hours. Its application is also recommended in typhoid fever, in cases where the patients manifest a dread of water, or where the application of water is impossible; likewise in other inflammatory diseases, especially the severer forms of rheumatism, and in tuberculosis.—*Giornale Veneto di Scienze Med., Ser.* 3, *Tom.* 21, 1874—*Allg. Med. Central-Zeitung,* 16*th Sept.,* 1874.

*Prolapsed Rectum.*—Surgeon C. Heath says: What I prefer myself is, to use nitric acid in stripes down the prolapsed rectum, rather than to smear the whole surface. If you adopt the latter plan, four or five stripes are sufficient, and thus all risk of producing too much contraction is avoided. Another mode of producing a cure is to narrow the anus. In these cases we find the anus has become distended by the habitual enlargement; and if you remove some of the skin about the margin of the anus by scissors and forceps, in the way I have already mentioned when speaking of hemorrhoids, the parts contract in healing, and a support is given to the lower end of the rectum, which practically cures the patient of his disease.

*New Antiseptic Ointment.*—Professor Lister, according to the *Student's Journal and Hospital Gazette,* is at present using, with great success, an ointment composed of paraffin, 2 parts; white wax, 1 part; sweet oil of almonds, 2 parts, and boracic acid (powdered), 1 part.—*Medical and Surgical Reporter.*

*Carbolic Acid in Tapeworm.*—Dr. S. A. Brown, U. S. N., writes to the New York *Medical Record,* March 16 : In a recent issue of your journal, I noticed a letter from Dr. Bills, of the army, recommending the use of carbolic acid in the treatment of tapeworm.

Having a case on hand at the time, which had resisted the use of oil of turpentine and pepo for a month or six weeks, I immediately administered to him the following prescription : ℞. Acid carbolic cryst., gr. ij.; glycer. pulv., gr. iv. Ft. pil., No. 2. To be taken every three hours, beginning at 9 A.M. and continued until 9 P.M. This followed in the morning by jalap and rhubarb, ten grains each. On the first day a few joints were passed ; on the second, a great number, and on the third, the head and narrow portion came away, with a great number of small sections, from one to three inches long. On the fourth day, treatment being kept up without further result, the medicine was discontinued.

The only sensation created in the stomach by the administration of such large quantities of the drug, was a slight burning, not sufficient to inconvenience the patient. I look upon this as a perfect cure, and therefore report the case to you.

*Hypodermic Injection of Morphia and Atropine.*—It is a good plan to combine one-thirtieth grain of sulphate of atropia with each one-quarter grain of morphia injected. This has a two-fold effect. (1) It diminishes the tendency to nausea so constantly following the administration of morphia alone. If more atropine is injected, symptoms of atropism show themselves—usually slight delirium or very rapid action of the heart. The effects of an over dose of atropine can be counteracted by the administration of more morphia.—*Dr. J. M. Finny.*

*Diphtheria.*—Dr. J. Lewis Smith (*N. Y. Medical Record*) says: The local treatment consists in the application to the fauces, every three hours, of the following, which has given me better satisfaction than any other medicine for local treatment which I have employed : ℞. Acidi carbolici, gtts. v.; liq. ferri subsulphat, ʒ ij.; glycerin, ʒ i. M. The brush was employed in preference to the probang, as it is less painful, and where there is a pseudo-membrane does not produce hemorrhage by its detachment.

*Formulas for Diarrhœa and Dysentery in Children.*—Dr. Q. C. Smith, Cloverdale, Sonoma Co., sends us (*Pacific Med. Jour.*) the two subjoined formulas, which he says he has found more reliable in the majority of cases than any of the prescriptions contained in the systematic treatises:

1. ℞. Fluid extract of ergot, ℥i.; fluid extract of golden seal, ℥ss. Mix. To small children, give from one-half to one teaspoonful at intervals of one to four hours, as the case may require. For dysentery, with bloody discharges.

2. ℞. Logwood chips, ℥ij.; powdered cinnamon bark, ℥j.; mix and put in a bottle with a pint of water, place the bottle in water and keep at a boiling heat for one hour, strain the liquor and bottle for use. Dose, from a teaspoonful to a tablespoonful every one, two, three or four hours, as the case may require. For diarrhœa of children caused by teething, warm weather, etc.

*Lentil Meal.*—Lentil meal, properly prepared and ground to a fine powder, and mixed with some other impalpable nitrogenous meal, such as rye-meal, possesses many valuable qualities. It is highly nitrogenous, and possesses four times the nutriment power of beef, weight for weight. It is very cheap—two and a half pounds cost twenty pence. The same quantity of the absurdly dear revalenta costs six shillings. It is so digestible that there is an absence of flatulence during the process.—*British Med. Jour.*

*Solution of Morphia for Hypodermic Injection.*—Dissolve ten grains of hydrochlorate of morphia in two drachms of distilled water by the aid of heat, without any acid, spirit or glycerin. Two minims of this solution, *i.e.* one-sixth of a grain, should be the commencing dose. It becomes solid at ordinary temperatures, and when wanted for use must be heated. The advantage is, that however long it is kept, the solution never spoils.—*Dr. H. Lawson.*

*Mammary Abcess (Threatened)—Application of Oleate of Mercury.* Apply a solution of oleate of mercury and morphia in oleic acid, simply brushed over the part. The mercury is rapidly absorbed and arrests the inflammatory action, the morphia at the same time relieving the pain.—*Mr. J. Marshall.*

*Use of Gelatin Suppositories in Obstinate Constipation due to Accumulation of Fœces in the Rectum.*—In the above cases, and when there exists an accumulation of hardened fæcal matter in the rectum or colon, Dr. Nagel finds that when purgatives and enemata have failed, and in order to dispense with the use of the anal *curette*, suppositories of gelatin constitute an easy, harmless and effective means of removing the evil. The suppositories are made of brown gelatin. They are steeped in water for twelve hours, and thus being softened and enlarged are introduced into the rectum. By subjecting the patient to a suitable regimen, an evacuation of pultaceous matter is obtained in twenty-four hours. The author attributes these effects to the hygrometic properties of the suppositories.—*Allgemeine Wiener Medical Zeitung.*

*Fatal Poisoning from Chlorate of Potassa.*—Dr. A. M. Ferris gives a case of supposed poisoning from chlorate of potassa, where a "large spoonful" of the salt was swallowed by mistake, and was followed by congestion of the surface, blue lips, cold extremities, inability to empty the bladder, hæmaturia, and death. At the post-mortem examination the auricles were found filled with coagula, and the ventricles empty and contracted. The lungs and abdominal viscera were healthy. As the brain and kidneys were not examined, absolute truth appears to be wanting that the symptoms were really due to the salt of potash.—*Pacific Medical nnd Surgical Journal.*

*Resemblance of Twins.*—Dr. Bird declares that, according to his own experience during thirty years, twins which are contained in the same membranes, and have a single placenta, resemble each other; while those which are contained in separate membranes, and have different placentæ, do not resemble each other more than other children of the same parents.—*La Tribune Medicale.*

*Hooping-Cough.*—Dover's powder is a remedy worth trying in hooping-cough. It should be given in doses of one grain thrice daily to a child twelve months old. It lessens the number and the severity of the attacks, and soothes the general irritability which so often exists.—*Dr. W. Maccall.*

*Catarrh.*—Dr. Channing calls attention to the danger of employing carbolic acid and ammonia in combination in the treatment of catarrh. The agents used separately (by inhalation) are frequently effectual in cutting short an incipient cold. The mixed solutions, however, produce decided narcotic symptoms, which the doctor explains on the hypothesis that aniline is formed by a reaction familiar to the chemist. A formula for an inhalation consisting of carbolic acid, ammonia, alcohol and water, originating in Berlin, has been quite extensively circulated in the medical journals. Dr. Channing has found carbolic acid alone so effectual that he thinks it hardly worth the while to risk the serious consequences which might follow from the addition of ammonia.—*Boston Journal of Chemistry.*

*To Check Vomiting from Cough.*—Consumptives and others suffering from paroxysms of cough frequently vomit their food in such paroxysms. Dr. Woiliez recommends swabbing the pharynx before eating with a concentrated solution of bromide of potash. ℞. Pot. brom., ʒij.; Aquæ destil., ʒiv.—warning the patient not to cough for a few minutes after the application. The same treatment is recommended for the vomiting of pregnancy. It is said to be very successful.

*Erysipelas—Styptic Colloid.*—Richardson's styptic colloid, painted over the affected surface twice in the twenty-four hours, both relieves the suffering and arrests the progress of erysipelas. It has, moreover, the additional merit of having an agreeable odor and being cleanly.—*British Medical Journal.*

PROFESSOR AUGUST VEGEL encloses ether in a gelatin capsule. In cases of tapeworm, the patient swallows a capsule, the ether is vaporized in the stomach, the worm is stupefied, and may be removed by the usual remedies, which are powerless when the worm is able to resist.—*Journal of Applied Chemistry.*

*Chloral and Ipecacuanha in Croup.*—In a bad case of croup with urgent dyspnœa, give to a child of fifteen months old two minims of ipecacuanha wine, with two grains of chloral, every two, three or four hours, according to the effect produced.—*Dr. G. Barclay.*

*Delirium Tremens after Chloral-Drinking.*—Dr. George F. Elliott relates an interesting case where the excessive use of chloral produced inaptitude for exertion, muscular pains in the upper extremities, loss of appetite, great thirst, constantly fetid breath, constipation, and finally a semi-comatose condition. The patient, a man æt. 35, had been in the habit of taking fifteen grains of opium daily for many years, but had for a few weeks substituted chloral, taking two hundred grains in the twenty-four hours. Its withdrawal produced at first sleepiness, and then all the phenomena of delirum tremens, which subsided under the use of large doses of tartar-emetic and opium.—*Lancet.*

*Enlargement of the Tonsils as a Cause of Nightmare.*—J. Warrington Haward, F. R. C. S., relates some cases of nightmare occurring in children, resisting ordinary methods of treatment, and increasing in frequency and severity, which were at once permanently cured by the removal of a portion of the tonsils. As these glands were enlarged, he was led to believe that the obstruction to respiration and consequent cerebral congestion were sufficient to produce the disorder. Nightmare due to gastric irritation or dentition occurs, as a rule, only once in the night, while that due to enlarged tonsils often recurs several times in the same night, and is invariably observed to be aggravated by the child catching cold.—*British Medical Journal.*

*Cholera.*—Dr. J. G. Sproston says: During the month of September I have prescribed for 150 cases, and have not had one fatal case. The plan I adopt is simple. To an adult I give an eight ounce mixture, composed as follows: ℞. Ac. sulph. dil., f ʒ ij.; syr. papaver., f ℥ iij., simply to color it; liq. ammon. acet., f ℥ ij.; aq. menth. pip., f ℥ ijss. M. Sig. f ℥ i. after every loose motion.

And very rarely had I to give a second bottle of medicine.—*Lancet.*

*Death from the Use of Perchloride of Iron.*—A foreign contemporary records a fatal instance of the use of a uterine injection of perchloride of iron. Peritonitis supervened soon after administration, and death occurred in thirty hours.—*Lancet.*

